# Hypoxia-driven macrophage heterogeneity in sepsis: single-cell transcriptomic insights into immune reprogramming and therapeutic targeting

**DOI:** 10.3389/fimmu.2026.1941002

**Published:** 2026-10-27

**Authors:** Yanfei Yu, Zhixing Wang, Yongxing Li, Cai Jing, Xia Li

**Affiliations:** 1 Department of Critical Care Medicine, The Affiliated Hospital of Qingdao University, Qingdao, China; 2 Department of Neurosurgery, The Affiliated Hospital of Qingdao University, Qingdao, China; 3 Dapartment of Pathology, The Affiliated Hospital of Qingdao University, Qingdao, China

**Keywords:** hypoxia, machine learning, sepsis, single-cell RNA sequencing, TIPARP

## Abstract

Sepsis is characterized by profound immune dysregulation and metabolic remodeling, yet how hypoxia shapes macrophage-state diversity remains incompletely defined. Here, we integrated single-cell transcriptomic profiles from early- and late-stage sepsis with healthy controls to construct a hypoxia-centered immune atlas. The single-cell landscape identified 103,851 cells across 23 clusters, while hypoxia scoring showed that macrophages displayed the strongest hypoxic activity, particularly in early sepsis. Macrophages were further stratified into low-, intermediate- and high-hypoxic states, revealing a continuous transcriptional gradient rather than discrete subsets. High-hypoxic macrophages exhibited increased CytoTRACE-defined plasticity, occupied intermediate-to-late pseudotime states, and showed enhanced inflammatory and metabolic pathway activation. Cell–cell communication analysis positioned these macrophages as bidirectional signaling hubs, with amplified MIF, CCL, ANNEXIN and IL1-related interactions. Integrated high dimensional Weighted Gene Co-expression Network Analysis (hdWGCNA) and machine-learning analyses identified hypoxia-associated hub genes, among which TIPARP emerged as a key regulator linked to macrophage hypoxic remodeling. TIPARP was preferentially enriched in high-hypoxic macrophages and experimentally validated as responsive to hypoxia and inflammatory stimulation, with knockdown can enhance HIF1A and VEGF induction. Drug-sensitivity prediction further suggested state-specific vulnerabilities of hypoxia-adapted macrophages. Together, these findings define hypoxia as a central determinant of macrophage heterogeneity in sepsis and nominate TIPARP as a candidate target for immunometabolic intervention.

## Introduction

Sepsis, a fatal syndrome induced by infection, is characterized by an imbalance in the host’s immune response and a significant remodeling of the microenvironment (1, 2). Despite the continuous advancements in diagnostic and therapeutic approaches in recent years, the high mortality rate of sepsis has still not been fundamentally improved (3–5). A growing body of evidence suggests that the functional heterogeneity and dynamic evolution of immune cells are the key determinants of the disease course and prognosis (5–7). However, traditional population - level transcriptomic studies are unable to clarify the fine regulatory mechanisms within the complex immune cell networks, which restricts the in - depth understanding of the underlying pathogenesis of sepsis (8, 9). The advent of single - cell RNA sequencing (scRNA-seq) technology offers an important opportunity to uncover the cell - type - specific transcriptional characteristics and their dynamic changes at different stages of the disease (10).

In the inflammatory microenvironment, hypoxia is considered as one of the crucial factors that drive the reprogramming of immune function (11, 12). Hypoxia not only regulates energy metabolism (13) but also influences the activation, differentiation and amplification of inflammatory responses via diverse signaling pathways (14, 15). Nevertheless, in the highly dynamic pathological process of sepsis, the mode of action of hypoxia in different immune cells and its influence on cell fate determination still lack systematic investigation (16, 17). Recent animal studies have further demonstrated that RIPK1/RIPK3/MLKL-mediated necrotic apoptosis and nitrosative stress are involved in the LPS-induced sepsis-induced cardiac and renal inflammatory injury and death, suggesting that microenvironmental stress and the inflammatory cell death program jointly contribute to organ dysfunction (18). Particularly for macrophages (19, 20), which are key effector cells, their core roles in inflammatory responses, antigen presentation and tissue repair have been widely acknowledged, yet the hierarchical characteristics of macrophage functional states driven by hypoxia and the molecular regulatory network have not been fully clarified (21).

Based on this foundation, this study integrated multi - cohort single - cell transcriptomic data to systematically construct a cellular map of sepsis, thereby revealing significant heterogeneity in the composition and transcriptional state of immune cells. Moreover, by utilizing a multi - algorithm scoring system, the hypoxic activity was quantified. It was discovered that macrophages in sepsis exhibited a significantly elevated hypoxic level and demonstrated a continuous gradient distribution of functional stratification characteristics. Additionally, the dynamic expression of certain key genes in the single - cell trajectory and their association with metabolic reprogramming further underscored the functional reshaping characteristics driven by hypoxia.

In conclusion, this study systematically unveiled the hypoxia - driven heterogeneity of macrophages and its core role in the immune microenvironment of sepsis from a single - cell resolution perspective. This not only enhanced the understanding of disease occurrence and development but also provided a novel theoretical basis for the targeted and precise intervention of immune metabolic reprogramming.

## Methods

### Data collection and processing

scRNA-seq data were retrieved from the Gene Expression Omnibus database (GEO, https://www.ncbi.nlm.nih.gov/geo/) and subsequently analyzed using the Seurat package in the R programming language (version 4.2.3). The objective of this study was to integrate the two datasets to investigate the dynamic changes during different stages of sepsis. The first dataset was obtained from patients diagnosed with sepsis within 0 to 6 hours (from GSE167363). The second dataset was derived from patients in the later stage of sepsis (diagnosed 14 to 21 days after the onset of sepsis), sourced from GSE175453. Furthermore, we also utilized the healthy control (HC) groups from these two datasets. For bulk RNA-seq validation, GSE95233 and GSE57065 were used as the discovery and validation cohorts, respectively.

Each single-cell dataset was initially processed independently to minimize dataset-specific technical variation. Cells with fewer than 200 or more than 7,000 detected genes, or with mitochondrial transcript proportions exceeding 10%, were excluded. Gene-expression matrices were normalized using the LogNormalize method with a scale factor of 10,000, and highly variable genes were identified using the vst method. To correct technical batch effects between the two independent GEO cohorts, we applied the Seurat anchor-based integration workflow. Briefly, 3,000 integration features were selected using SelectIntegrationFeatures, integration anchors were identified using FindIntegrationAnchors based on the first 20 dimensions, and the datasets were subsequently integrated using IntegrateData with dims = 1:20. Dataset origin was treated as the technical batch variable during integration, whereas disease-stage annotations were retained for subsequent biological comparisons and were not regressed out.

Principal component analysis (PCA) was subsequently performed on the integrated expression matrix. The first 20 principal components were used to construct the shared nearest-neighbor graph, followed by unsupervised clustering with a resolution of 0.5. Uniform Manifold Approximation and Projection (UMAP) was used for visualization. Cell identities were assigned according to established canonical marker genes.

### Identification of high-hypoxic cells

The five hypoxia activity scores derived from AUCell, UCell, singscore, ssGSEA, and AddModuleScore were standardized, min-max normalized to the 0–1 range, and summed to obtain a composite hypoxia score. Macrophages were stratified using an approximately equal-frequency tertile strategy based on the 33rd and 66th percentiles of the composite score. The exact cutoffs were 2.4096 and 2.8856, yielding LHM (score < P33; n = 5,442), IHM (P33 <= score < P66; n = 5,442), and HHM (score >= P66; n = 5,607). These groups were treated as operational analytical states along a continuous hypoxia gradient rather than naturally discrete cell subpopulations or clinically validated thresholds.

### Pseudo-time trajectory analysis and intercellular interactions analysis

Trajectory analysis is based on the objects pre - processed by Seurat. It utilizes SeuratWrappers to establish a connection with Monocle3 (22), constructs a k-nearest neighbor (KNN) graph in the UMAP, and learns the main trajectory, computes the pseudotime and branching structure. Subsequently, the built - in models of Monocle3 are employed to assess the dynamic changes of genes with respect to the pseudotime, and cell to cell communication is carried out using CellChat (23). Based on the expression matrix of annotated cell types and the ligand-receptor database, significant interactions are deduced, and the sending/receiving intensity and pathway contribution are calculated. Through permutation tests and multiple corrections to control false positives, and the ggplot2 package is used for network and pathway visualization.

### HdWGCNA

We employed hdWGCNA (24) to construct gene co-expression networks and identify modules associated with high-hypoxic macrophages (HHM). After quality-control filtering, metacells were generated to reduce sparsity in the single-cell expression matrix and improve the robustness of correlation estimation. A weighted gene co-expression network was subsequently constructed, and a soft-thresholding power of β = 6 was selected according to the scale-free topology criterion. The adjacency matrix was transformed into a topological overlap matrix (TOM), followed by hierarchical clustering and dynamic tree cutting to identify co-expression modules. Modules with highly similar module eigengenes (MEs) were subsequently merged. Module eigengene-based connectivity (kME) was calculated to quantify the membership of each gene within its corresponding module. Genes with kME ≥ 0.30 were retained as module-associated hub genes for subsequent analyses, thereby excluding genes with weak module membership. Finally, module eigengenes were correlated with macrophage hypoxic states to identify modules positively associated with the HHM phenotype.

### Select the characteristic genes of HHM

We employed four machine-learning strategies to identify characteristic genes associated with HHM. The Least Absolute Shrinkage and Selection Operator (LASSO) (25)was first applied for feature selection using an iterative reweighted least-squares approach, with optimal variables determined after 1,000 iterations based on the minimum criterion. In parallel, univariate Cox regression analysis was conducted to screen candidate genes, and those with P < 0.05 were retained for further evaluation. Random Forest (RF) (26) was subsequently implemented owing to its strong predictive performance, robustness to overfitting, and ability to quantify feature importance, making it particularly suitable for high-dimensional datasets. To further refine feature selection, the Boruta algorithm was applied as a supervised wrapper method to identify all relevant variables by iteratively comparing observed features against randomized counterparts, thereby improving selection reliability. In addition, a Decision Tree (DT) (27) model based on the Classification and Regression Tree (CART) algorithm was constructed, with model complexity optimized through cross-validation by tuning tree depth and pruning parameters to achieve optimal feature partitioning. Finally, Adaptive Best Subset Selection (ABESS) (28) was used to identify the most parsimonious set of predictors within a sparse modeling framework.

### Machine learning benchmarking for HHM features

To identify the optimal predictive model for HCEM features at the single-cell level, we systematically evaluated eight machine learning algorithms using the mlr3 R framework. The evaluated models included logistic regression (LR), KNN, linear discriminant analysis (LDA), naive Bayes (NB), random forest (Ranger), recursive partitioning and regression trees (RPART), support vector machines (SVM), and extreme gradient boosting (XGBoost). Cells annotated as HHM were defined as positive samples, whereas LowStem cells were used as controls. The dataset was randomly partitioned into training (80%) and test (20%) subsets. Hyperparameters were optimized using five-fold internal cross-validation, and model performance was further assessed through ten-fold external cross-validation to ensure generalizability. Model performance was quantified using the area under the receiver operating characteristic curve (AUC), and the algorithm with the highest mean AUC was selected as the optimal model for predicting the HHM signature. Fold-wise AUC values obtained from identical repeated five-fold cross-validation partitions were treated as paired measurements. Normality was examined using the Shapiro–Wilk test, and variance homogeneity was assessed using the Fligner–Killeen test. Overall differences among the eight classifiers were evaluated using the Friedman rank test with resampling iteration as the blocking factor, followed by two-sided paired Wilcoxon signed-rank tests with Holm adjustment for 28 pairwise comparisons. A Nadeau–Bengio corrected repeated-cross-validation test was additionally performed as a sensitivity analysis to account for dependence among overlapping cross-validation training sets.

### Cell culture

RAW264.7 macrophage cells (passages 3–6) were purchased from Haixing Biosciences (Suzhou, China;TCM-C766). Cells were maintained in Dulbecco’s Modified Eagle Medium (DMEM) supplemented with 10% fetal bovine serum(FBS) and 1% penicillin–streptomycin, and cultured at 37 °C in a humidified incubator with 5% CO_2_ until use.

### Western blot

Tissues or cells were lysed on ice in RIPA buffer (Thermo Fisher, 89900). Proteins were quantified by BCA assay (Thermo Fisher, 23227), separated by SDS–PAGE, and transferred to 0.45 µm PVDF membranes. Membranes were blocked in TBST with 5% nonfat milk for 1 h, incubated overnight at 4°C with primary antibodies—TIPARP (1:1000, Abcam ab200390); HIF1A (1:1000,Proteintech 39666); VEGF (1:10000, 19003-1-AP);GAPDH (1:20000, 10494-1-AP)—and then with HRP-conjugated secondary antibodies (Proteintech RGAM001 or RGAR001) for 1 h at room temperature. The electrochemiluminescence (ECL) test kit (Beyotime, P0018S) was employed to identify protein bands on a developer after five 10-min washes with TBS-T. The ImageJ software was utilized to calculate the protein bands, and the results were normalized with respect to GAPDH.

### Quantitative real-time PCR

Total RNA was extracted from cultured cells and matched tissue samples using TRIzol reagent (Invitrogen, 15596026) according to the manufacturer’s instructions. cDNA was synthesized from 1 µg total RNA using a qPCR kit (Vazyme, China). RT-qPCR was performed on a StepOnePlus Real-Time PCR System (Applied Biosystems) with Fast SYBR Green Master Mix (Thermo Fisher Scientific) under recommended conditions; melt curve analysis confirmed single-product amplification. Primer pairs were: *TIPARP* forward 5′-CAGCCCTACAGCAACAACCT-3′ and reverse 5′-GTCCTTGGCATCTGGTTGTC-3′; *HIF1A* forward 5′-AGCAATTCTCCAAGCCCTCC-3′ and reverse 5′-TTCATCAGTGGTGGCAGT TG-3′; *VEGF* forward 5′-GGGAGCAGAAAGCCCATGAA-3′ and reverse 5′-AGATGTCCACCAGGGTCTCA-3′; *GAPDH* forward 5′-GCATT GCTGACAATCTTGAGTGAG-3′ and reverse 5′-TGTCTTCACCACCATGGAGAAG-3′. Each reaction was run in technical triplicate, and experiments were repeated independently at least three times. Relative mRNA abundance was calculated using the 2^−ΔΔCt^ method with GAPDH as the internal reference; expression in the control group was set to 1.

## Results

### Single cell landscape of sepsis

Using single - cell sequencing data, an evaluation was conducted on the differences between normal samples and sepsis samples. A total of 103,851 cells were incorporated into the analysis (**Figure 1A**). The UMAP plot demonstrates that cells are distinctly clustered into multiple transcriptionally - defined groups. In the integrated dataset, cells are segregated into 23 clusters corresponding to the principal cell types (**Figure 1B**). By utilizing the established marker genes to annotate the cell clusters, diverse groups were recognized, including NK cells, B cells, macrophages, neutrophils, endothelial cells, and epithelial cells, among others (**Figure 1C**). To evaluate the disparities in cell components, **Figure 1D** quantified the relative proportions of each cell type across samples, and this heterogeneity implies potential differences in the sample - specific immune microenvironment. The heatmap of representative marker genes further corroborates the annotation results of cell types (**Figure 1E**). Distinct expression patterns were observed in each cluster, with genes possessing specific lineage characteristics being abundant in the corresponding cell populations. The feature map depicts the spatial expression patterns of the selected genes in the UMAP embedding space (**Figure 1F**). These genes display specific expression characteristics for the clusters, which are in line with their well - known biological functions.

**Figure 1:**
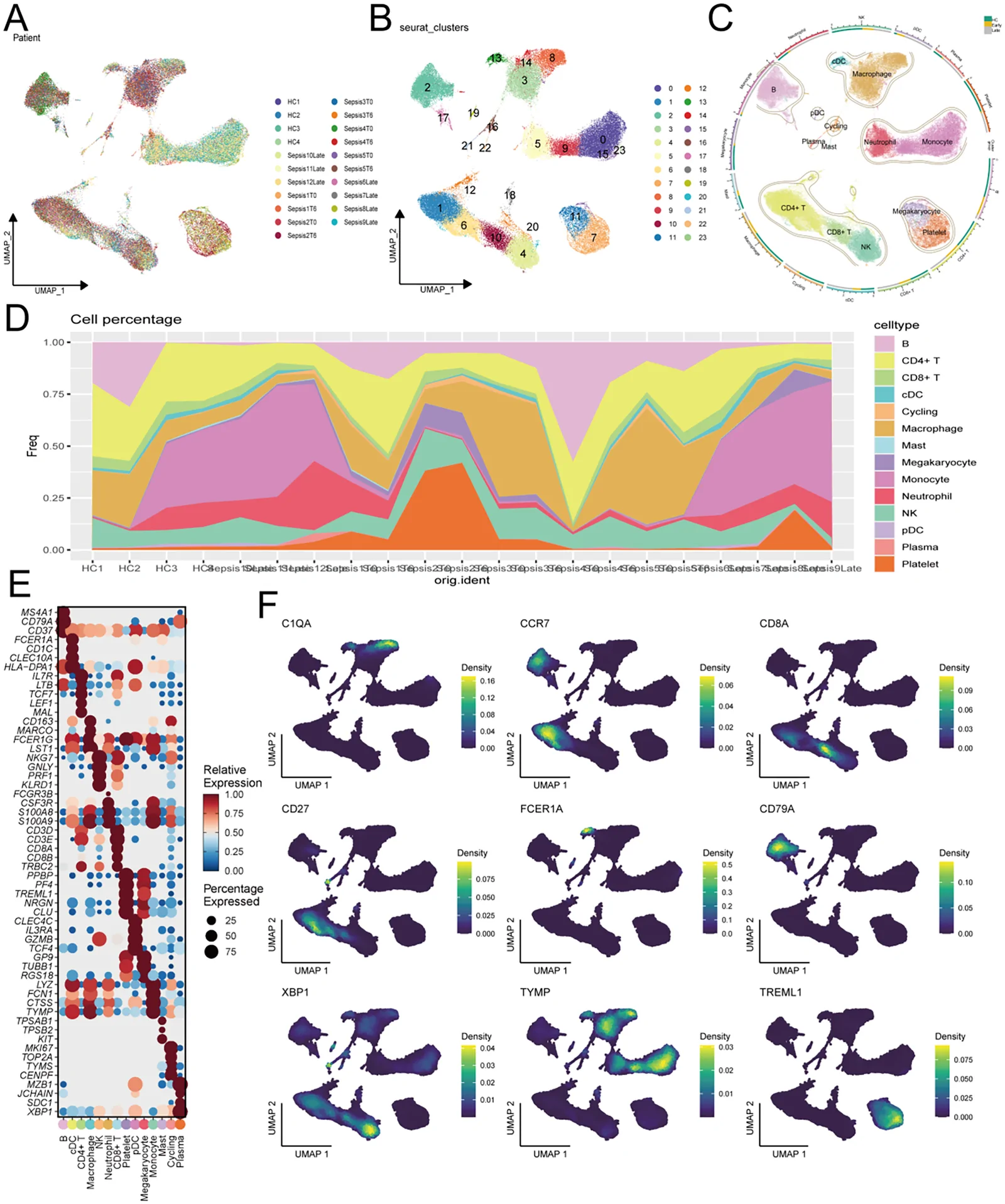
Single-cell transcriptome analysis revealed the cell heterogeneity among samples and different cell populations. **(A)** The UMAP profiles of 21 samples. **(B)** Using unsupervised clustering, all the cells were visualized using UMAP, and 24 distinct cell clusters were identified. **(C)** UMAP based on typical marker genes (including epithelial cells, immune subsets and stromal populations) annotated by major cell types. **(D)** The relative proportions of each cell type within the sample. **(E)** The heat map depicting the expression patterns of representative marker genes specific to cell types across clusters, which are utilized for cluster annotation. **(F)** The feature map depicts the expression of the selected marker genes and is projected onto the UMAP to validate the cluster - specific gene expression patterns.

### Evaluate the hypoxia activity of various cell types

We identified a cohort of genes associated with hypoxia from public databases and published papers, amounting to 396 genes in total (**Supplementary Table 1**). To systematically assess the hypoxic activity in sepsis samples at the single - cell level, we initially computed the hypoxic scores of sepsis samples and healthy samples. The outcomes indicated that the hypoxic activity of sepsis samples was notably higher than that of healthy samples across various large - scale RNA - seq datasets (**Figure 2A**). Subsequently, we employed a set of meticulously selected hypoxic genes and evaluated the hypoxic activity of cell types in sepsis samples via four distinct scoring methods (AUCell, UCell, singscore, ssGSEA, and Add). The scatter plot demonstrated that the expression of hypoxia - related genes in macrophages was relatively higher when compared to other cell types (**Figure 2B**). The bar graph illustrating the distribution of cell type scores also verified that the hypoxic activity of macrophages was significantly elevated (**Figure 2C**). The results of these four scoring methods were in agreement (**Figure 2D**). A further comparison of early sepsis samples, late sepsis samples, and healthy samples disclosed that the hypoxic activity of macrophages was significantly increased (**Figure 2E**). The bar graph depicting the distribution of hypoxic scores for different cell types showed that the hypoxic activity score of macrophages was significantly higher than that of the majority of other cell types (**Figure 2F**). **Figure 2G** presents the UMAP (Unified Mapping Projection) plot of hypoxic scores for different cell types. This UMAP plot visually depicts the hypoxic activity of early sepsis samples, late sepsis samples, and healthy samples, with the hypoxic activity of early sepsis being significantly higher (**Figure 2H**).

**Figure 2:**
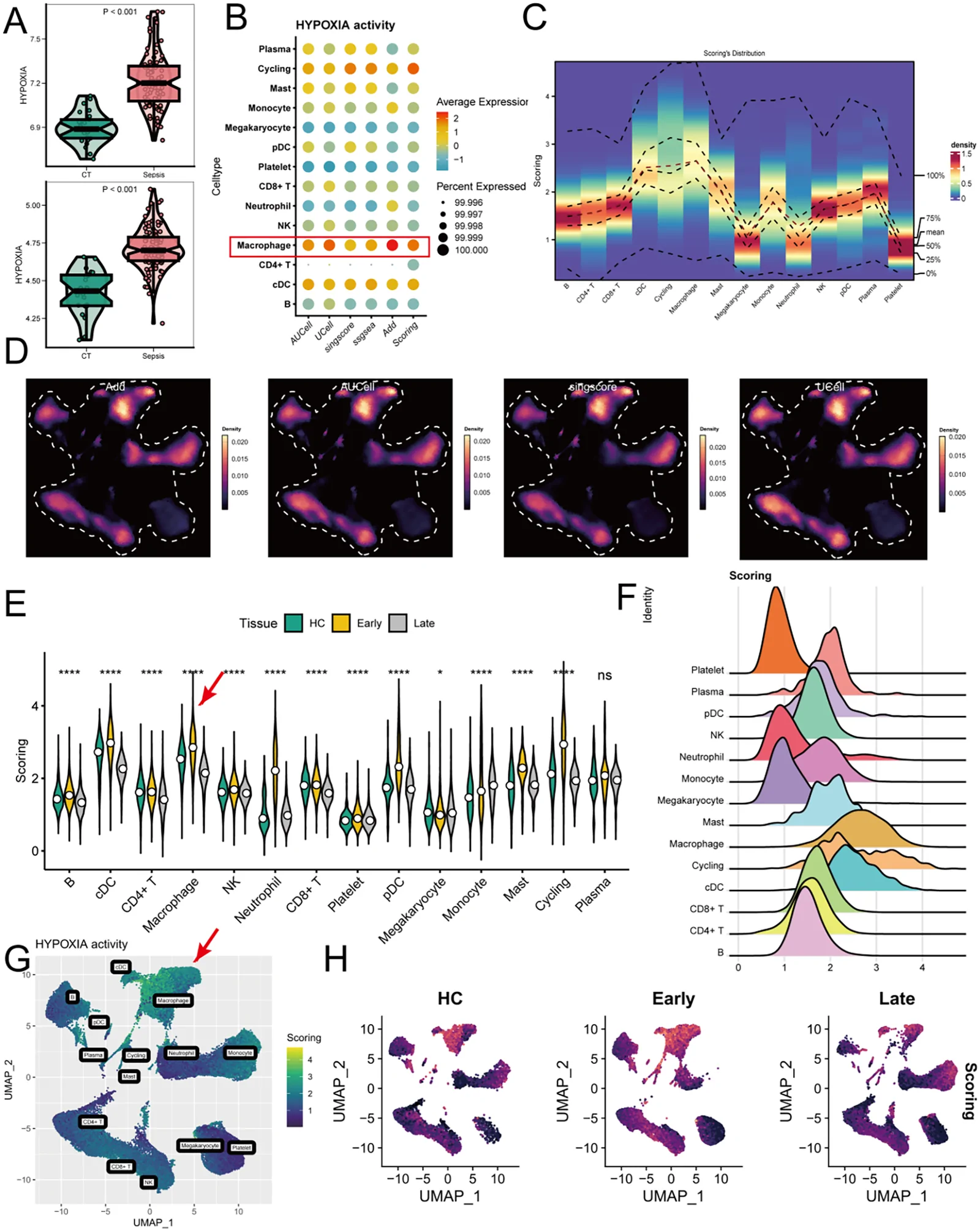
Hypoxia-related gene signatures reveal elevated hypoxic activity in macrophages during sepsis at the single-cell level. **(A)** Comparison of hypoxic scores between sepsis and healthy samples across multiple bulk RNA-seq datasets, demonstrating significantly increased hypoxic activity in sepsis. **(B)** Scatter plot showing hypoxia-related gene expression across major cell types, indicating relatively higher expression in macrophages. **(C)** Bar plot illustrating the distribution of hypoxic scores among different cell types, confirming elevated hypoxic activity in macrophages. ^*^p < 0.05, ^****^p < 0.0001. **(D)** Concordance analysis of four hypoxia scoring methods (AUCell, UCell, singscore, ssGSEA, and Add), showing consistent evaluation results. **(E)** Comparative analysis of hypoxic activity among early sepsis, late sepsis, and healthy samples, highlighting a significant increase in macrophages during sepsis progression. **(F)** Bar plot displaying hypoxic score distributions across cell types, with macrophages exhibiting the highest scores relative to other populations. **(G)** UMAP visualization of hypoxic scores across all cell types, illustrating spatial heterogeneity of hypoxic activity. **(H)** UMAP comparison of early sepsis, late sepsis, and healthy samples, demonstrating markedly elevated hypoxic activity in early sepsis.

### Identification of hypoxic activity of macrophages in sepsis

Based on the distribution of hypoxia activity scores in macrophages, cells were stratified into three distinct groups: the LHM group, comprising cells with scores below 2.409 (approximately the lowest 33%); the IHM group, including cells with scores between 2.409 and 2.885; and the HHM group, consisting of cells with scores above 2.885 (approximately the highest 66%). This stratification reflects a gradient of hypoxic activity, with 5,442 cells assigned to both the LHM and IHM groups and 5,607 cells categorized into the HHM group, enabling subsequent analyses of hypoxia-associated functional heterogeneity (**Figure 3A**). Sensitivity analyses supported the robustness of the percentile-based stratification. When analyzed as a continuous variable, the composite hypoxia score was positively associated with CytoTRACE (Pearson r = 0.547; Spearman rho = 0.544). Moreover, the HHM-LHM CytoTRACE contrast remained directionally consistent across median, P40/P60, P33/P66, P30/P70, P25/P75, P20/P80, and P10/P90 definitions. Under the P33/P66 definition, the standardized difference was 1.224 SD (sample-cluster bootstrap 95% CI, 0.878-1.458), indicating that the observed gradient was not dependent on a single cutoff choice (**Supplementary Figures S1A–D**). The grouped UMAP plot demonstrates that HHM cells are predominantly distributed in the middle to right regions of the map. LHM cells are more concentrated in the left and peripheral areas, and IHM cells are extensively distributed between the two, exhibiting a continuous transition from LHM to HHM (**Figure 3B**). The UMAP density plot further reveals that the low oxygen score exhibits a significant spatial heterogeneity distribution within the macrophage population. The high - score areas are mainly enriched in the right and central cell subgroups, whereas the overall score in the left area is relatively low, suggesting that there is a significant low - oxygen functional stratification in different spatial clusters (**Figure 3C**). The box plot quantifies the Cytytotrace scores of HHM, IHM and LHM (**Figure 3D**). The cells display a continuous rather than discrete distribution within the UMAP space, which implies significant transcriptional transition relationships among different cell populations. The CytoTRACE analysis reveals that cells with higher CytoTRACE values are predominantly concentrated in the upper and middle regions of the UMAP, whereas cells with lower CytoTRACE values are more prevalently distributed along the bottom edge, thereby forming a continuous gradient from high to low. Moreover, when the custom Scoring is overlaid with CytoTRACE, it is observable that the spatial distributions of the two are generally consistent. The regions with high hypoxia scores exhibit a high degree of overlap with the regions of high CytoTRACE, which supports the hypoxic macrophages exhibit a higher level of transcriptional complexity and are in a state related to metabolic/inflammatory activation. (**Figures 3E, F**). Meanwhile, the density maps of LHM, IHM and HHM indicate that the high - signal hotspots gradually expand from a focal area to a broader region. In particular, the HHM group exhibits a larger and more intense enrichment range, which implies that with alterations in the hypoxic state, the related cell states experience systematic remodeling (**Figure 3G**). **Figure 3H** demonstrates that the low-oxygen-related score of macrophages exhibits a significant positive correlation with the cell developmental potential. The scatter plot reveals a moderate positive correlation between the scoring and CytoTRACE (Pearson r = 0.55, 95% CI 0.54–0.56, P < 0.001), indicating that an increase in low-oxygen activity is associated with a higher cell state plasticity or dedifferentiation potential.

**Figure 3:**
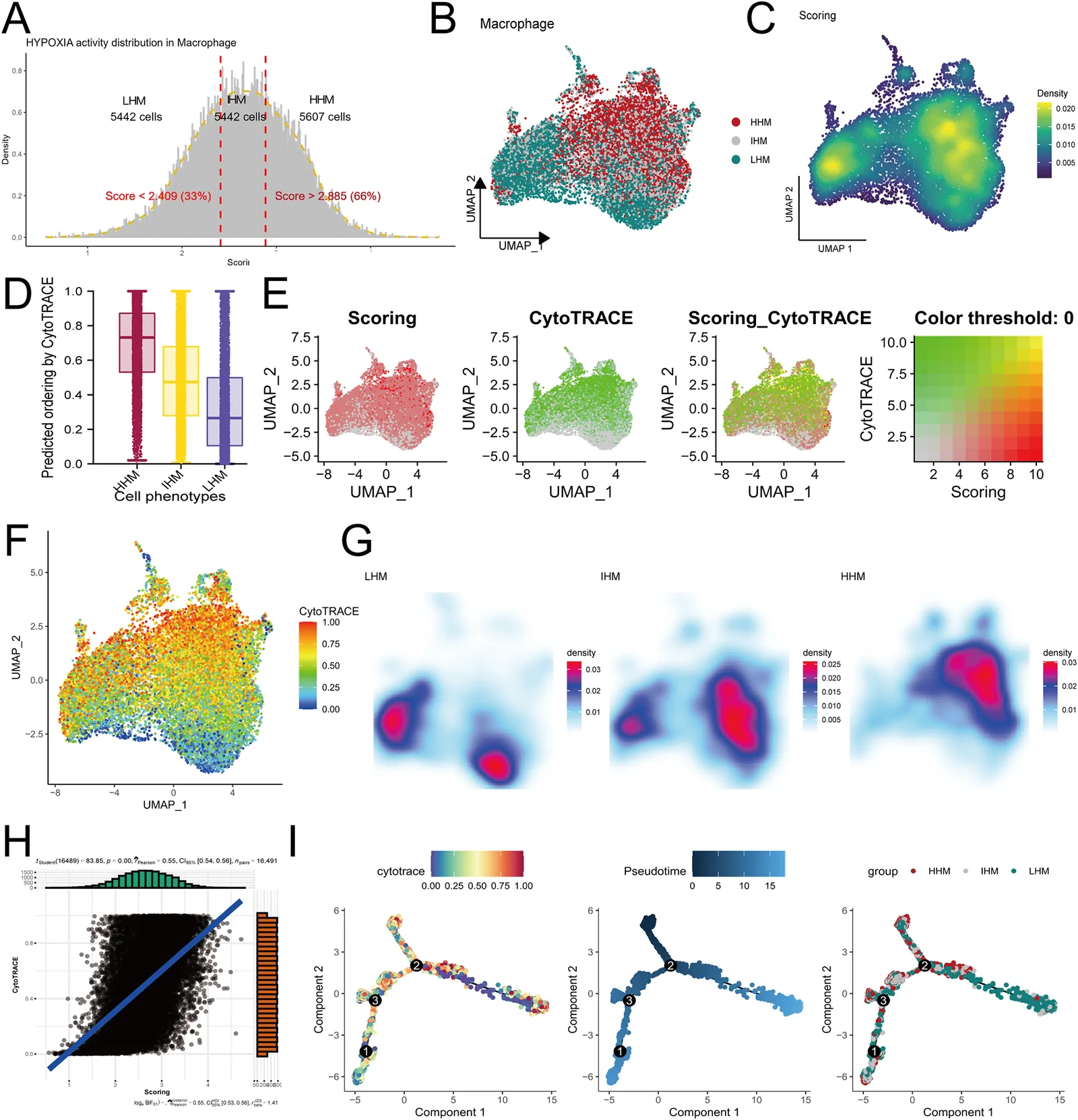
Hypoxia-based stratification reveals functional heterogeneity and developmental dynamics of macrophages. **(A)** Macrophages were stratified into three groups based on hypoxia scores: LHM, IHM and HHM, reflecting a gradient distribution of hypoxic activity. **(B)** UMAP visualization showing the spatial distribution of LHM, IHM and HHM cells, with HHM enriched in the middle-right regions, LHM localized to peripheral/left regions and IHM distributed between them, indicating a continuous transition. **(C)** UMAP density plot illustrating spatial heterogeneity of hypoxia scores, with high-score regions enriched in central and right clusters and low-score regions in peripheral areas. **(D)** Box plot comparing CytoTRACE scores among LHM, IHM and HHM groups, quantifying differences in developmental potential. **(E, F)** UMAP overlays of CytoTRACE and custom hypoxia scoring, demonstrating consistent spatial distributions and a strong overlap between high hypoxia scores and high CytoTRACE values. **(G)** Density maps of LHM, IHM and HHM groups. **(H)** Correlation analysis between hypoxia scores and CytoTRACE values. **(I)** Single-cell trajectory analysis depicting differentiation dynamics under hypoxic gradients, with LHM cells enriched at early stages and HHM cells distributed in middle-to-late stages along pseudotime, forming distinct developmental branches.

Single cell trajectory analysis unveiled the developmental dynamics of macrophages under diverse hypoxic conditions. In the Cytotrace scoring graph, various cells exhibited distinct branching structures, which indicated the heterogeneity of developmental potential. HHM was predominantly distributed in the middle and later phases of the trajectory, whereas LHM was mostly clustered at the starting end. Pseudo - time analysis further elucidated the continuous dynamics of cell differentiation, progressing from the initial state (node 1) to the mature state (node 3) and forming three principal branches. This suggests that cells possess different developmental pathways under the oxidative gradient. The trajectory graphs marked by groups demonstrated that HHM, IHM, and LHM cells displayed a gradient distribution along the pseudo - time axis. Hyperoxic cells were dominant in the middle and later stages, while hypoxic cells were at the initial stage, reflecting the substantial influence of the oxidative state on the differentiation trajectory of macrophages (**Figure 3I**).

### Analysis of intercellular communication among various cells in sepsis

Analysis of the intercellular communication network demonstrates that there exist highly intricate interactions among diverse immune cells and subsets of macrophages. In the interaction network diagram, HHM, monocytes and CD4+ T cells exhibit the largest nodes, which implies their central position in signal transmission. HHM cells engage in the most frequent and active communication with Cycling, cDC, Mast and pDC cells. The analysis of interaction intensity further reveals that the signal output and input of HHM cells are significantly higher compared to those of LHM cells (**Figure 4A**). The heatmap of outgoing signaling patterns demonstrates that HHM exhibits a robust outward output capacity across multiple signaling pathways, including MIF, GALETTIN, RESISTIN, ANNEXIN, CCL, VISFATIN, and IL1. This implies that HHM may serve as a crucial signal sender in the remodeling of the microenvironment. Simultaneously, the incoming signaling patterns indicate that HHM also displays a high response intensity to diverse ligand signals, reflecting its dual function of notable signal reception (**Figure 4B**). The ligand - receptor interactions between macrophages and various immune cells in the HHM state were also notably enhanced, indicating that macrophages occupy a more active regulatory position in the cell communication network. The interaction patterns of the receptors demonstrated that the interactions between macrophages and Cycling cells, cDC, Mast cells, and Monocytes in the HHM group were the most prominent, reaching 10, 8, 6 and 5 pairs respectively, all of which were higher than those in the LHM group. The overall log2(HHM/LHM) values were generally positive, suggesting that macrophages in the hypoxic and hyperoxic states are more prone to act as signal - receiving hubs in the microenvironment response. Further analysis of the interaction patterns of the provided ligands indicated that Mast cells, Cycling cells, and cDC were the primary signal - sending sources for macrophages. The corresponding interaction pairs in the HHM group reached 14, 13 and 10 respectively, significantly exceeding those in the LHM group **Figure 4C**. Further analysis reveals that the HHM sub - population possesses a higher outgoing interaction strength and incoming interaction strength, suggesting that it assumes a hub - like role in the intercellular communication network (**Figure 4D**). **Figure 4E** demonstrates that the intercellular communication centered around different hypoxic states of macrophages presents distinct directional and hierarchical characteristics. As signal transmitters, HHM, IHM, LHM can all establish extensive interactions with B cells, T cells, etc. Nevertheless, the pathways associated with HHM are more numerous, primarily encompassing ANXA1–FPR1/FPR2, CCL3–CCR1, IL1B–IL1R2, etc., indicating that HHM macrophages possess a stronger potential for inflammatory amplification and immune regulation. As signal recipients, macrophages mainly receive stimulation from CD4+ T cells, CD8+ T cells, cDC, etc. Among these, the MIF–CD74+CD44, ANXA1-FPR1, IL16-CD4, CCL5/CCL3–CCR1, and MDK-related axes are the most widely distributed. Moreover, HHM has more corresponding interaction points and a higher probability of communication. Overall, HHM macrophages exhibit both robust signal output and input characteristics within the cell communication network and may serve as the key hub in shaping the pro - inflammatory hypoxic microenvironment. The GSVA analysis indicated that multiple hallmark pathways in the HHM group were overall upregulated, among which hypoxia and TNFA_SIGNALING_VIA_NFKB were the most prominent (**Figure 4F**).

**Figure 4:**
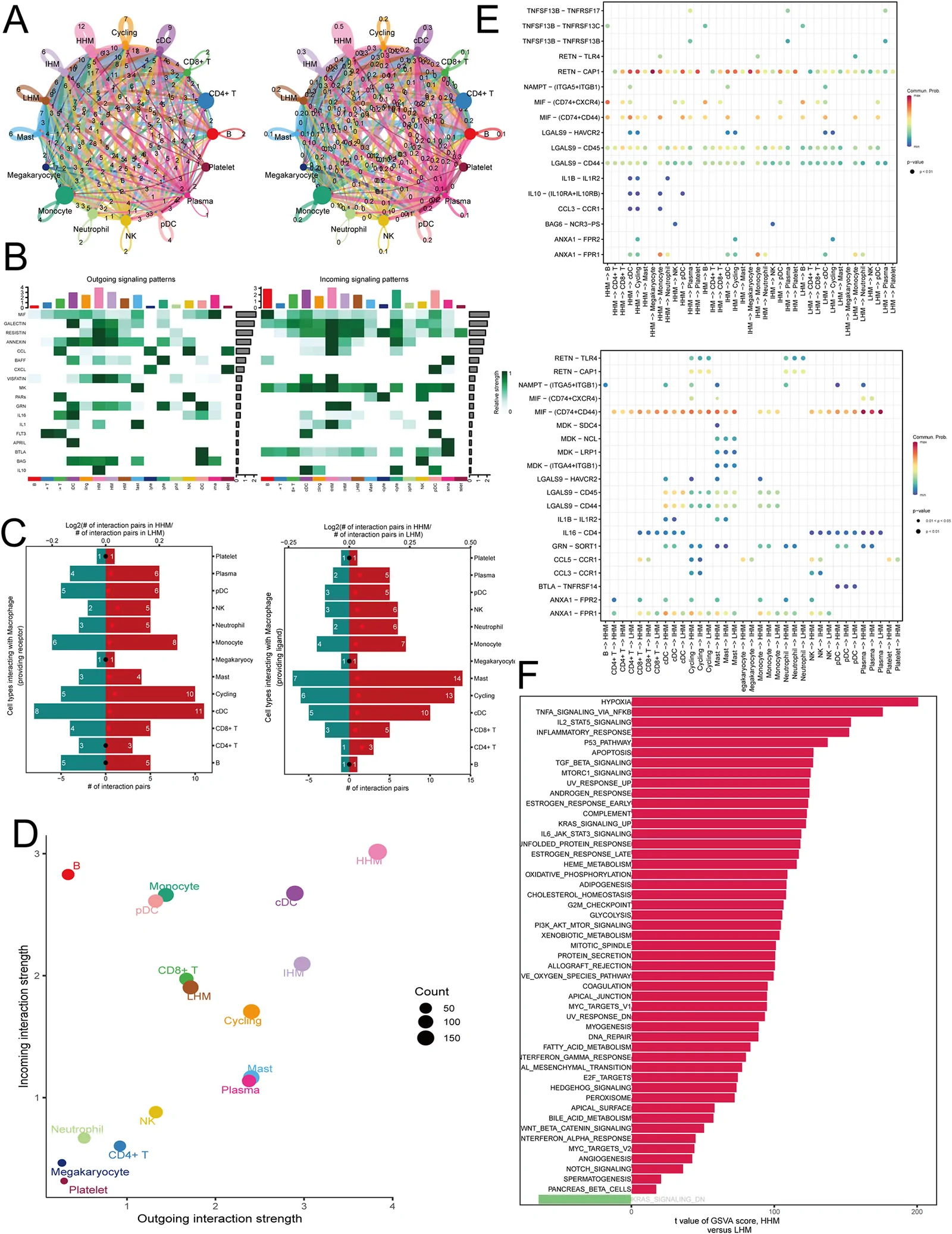
Intercellular communication analysis highlights the central regulatory role of HHM macrophages in the hypoxic microenvironment.**(A)** The global intercellular communication network. **(B)** Heatmaps of outgoing and incoming signaling patterns across macrophage subsets. **(C)** Quantification of ligand–receptor interaction pairs between macrophages and other immune cells. **(D)** Comparison of overall outgoing and incoming interaction strength among macrophage subsets. **(E)** Hierarchical and directional intercellular communication networks centered on macrophage subsets. **(F)** GSVA enrichment analysis of hallmark pathways.

### Identification of co - expressed gene modules associated with HHM via hdWGCNA

We employed hdWGCNA to identify the co-expression gene modules associated with HHM. An unscaled co-expression network was constructed, and an optimal soft threshold of 6 was selected (**Figures 5A, B**). We identified eight distinct gene co-expression modules (**Figures 5C, D**), among which the pink, red, brown, blue and yellow modules were highly activated in HHM (**Figure 5E**). Further investigations were carried out on the correlations between each module (**Figure 5F**). Within these HHM-associated modules, genes with module eigengene connectivity (kME) ≥ 0.30 were retained as module-associated hub genes for downstream analyses. The corresponding protein–protein interaction networks of the five HHM-associated modules are presented in **Figure 5G** and **Supplementary Table 2**.

**Figure 5:**
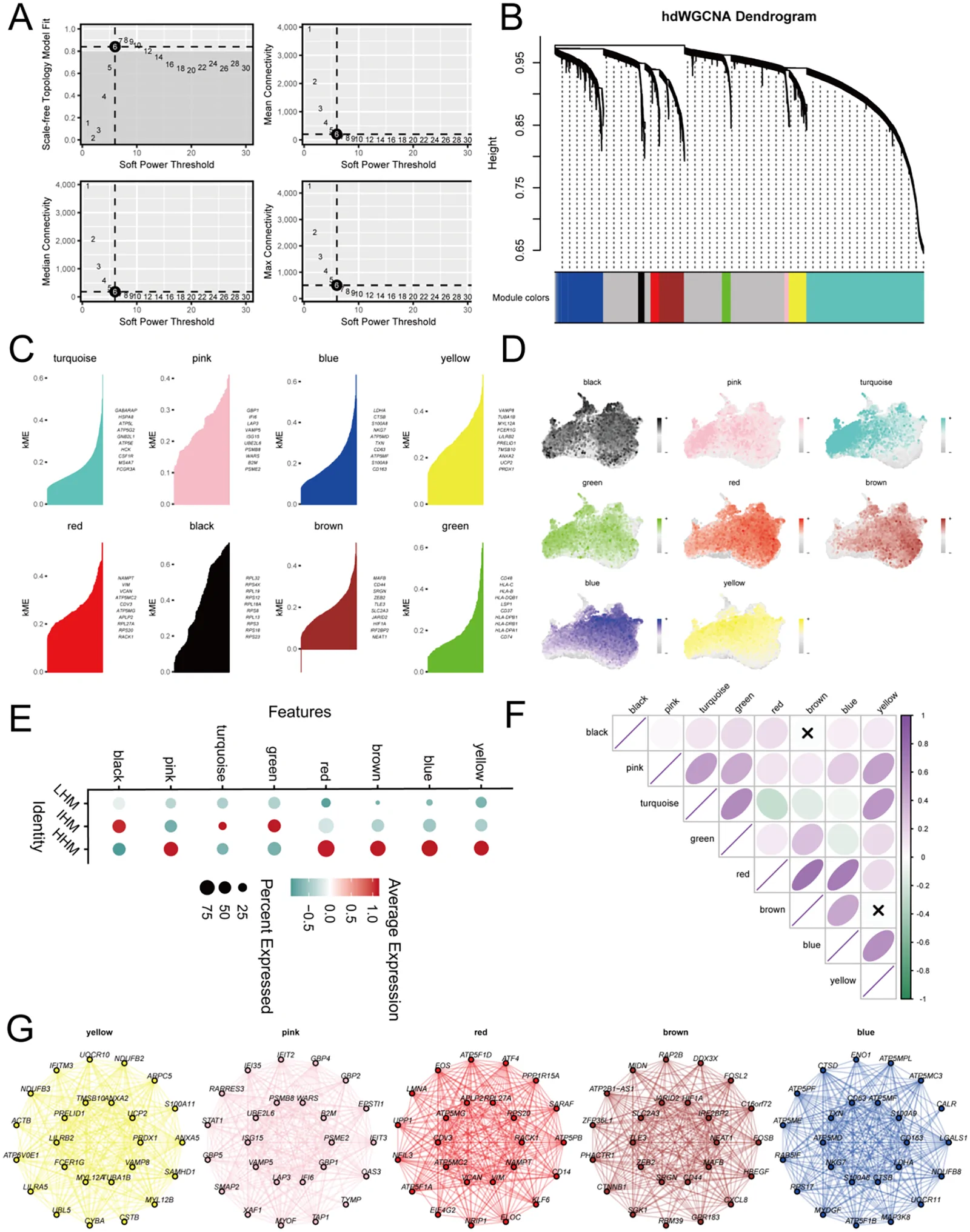
hdWGCNA identifies HHM-associated gene co-expression modules and their interaction networks. **(A)** Determination of the optimal soft-thresholding power for network construction, with a value of 6 selected to ensure scale-free topology. **(B)** Construction of the gene co-expression network based on hdWGCNA analysis. **(C)** Identification of distinct gene co-expression modules, each represented by different colors. **(D)** Hierarchical clustering dendrogram displaying the distribution of genes across modules. **(E)** Module activity analysis showing that the pink, red, brown, blue and yellow modules are highly enriched in the HHM group. **(F)** Correlation heatmap illustrating the relationships among different gene modules. **(G)** PPI networks of key modules, highlighting hub genes and interaction patterns.

### Utilize machine learning algorithms to conduct further screening for hub HRGs.

Differential expression analysis between the HHM and LHM groups revealed that 417 genes were upregulated (**Figure 6A**; **Supplementary Table 3**). Then, the intersection was taken with the 492 genes identified from the five hdWGCNA modules that had the strongest positive correlation with the HHM phenotype (**Supplementary Table 4**), resulting in 222 genes being simultaneously recognized as HRGs and differentially expressed genes (DEGs). This highlights their crucial role in the upregulation process under hypoxic conditions (**Figure 6B**; **Supplementary Table 5**). Pearson correlation analysis identified 192 genes that exhibited a significant positive correlation with hypoxia activity in HHM cells (correlation coefficient > 0 and adjusted P value < 0.05), with detailed results shown in **Figure 6C** and **Supplementary Table 6**. The Disease Ontology (DO) bubble plot highlights the diseases most strongly associated with the specific gene sets based on “gene ratio” and “P value,” revealing a marked correlation between hepatitis and the analyzed genes (**Figure 6D**; **Supplementary Table 7**). The Gene Ontology (GO) bubble plot further explores the biological processes, cellular components, and molecular functions related to the genes. Notably, strong associations were found with neutrophil activation and neutrophil-mediated immunity (**Figure 6E**; **Supplementary Table 8**).

**Figure 6:**
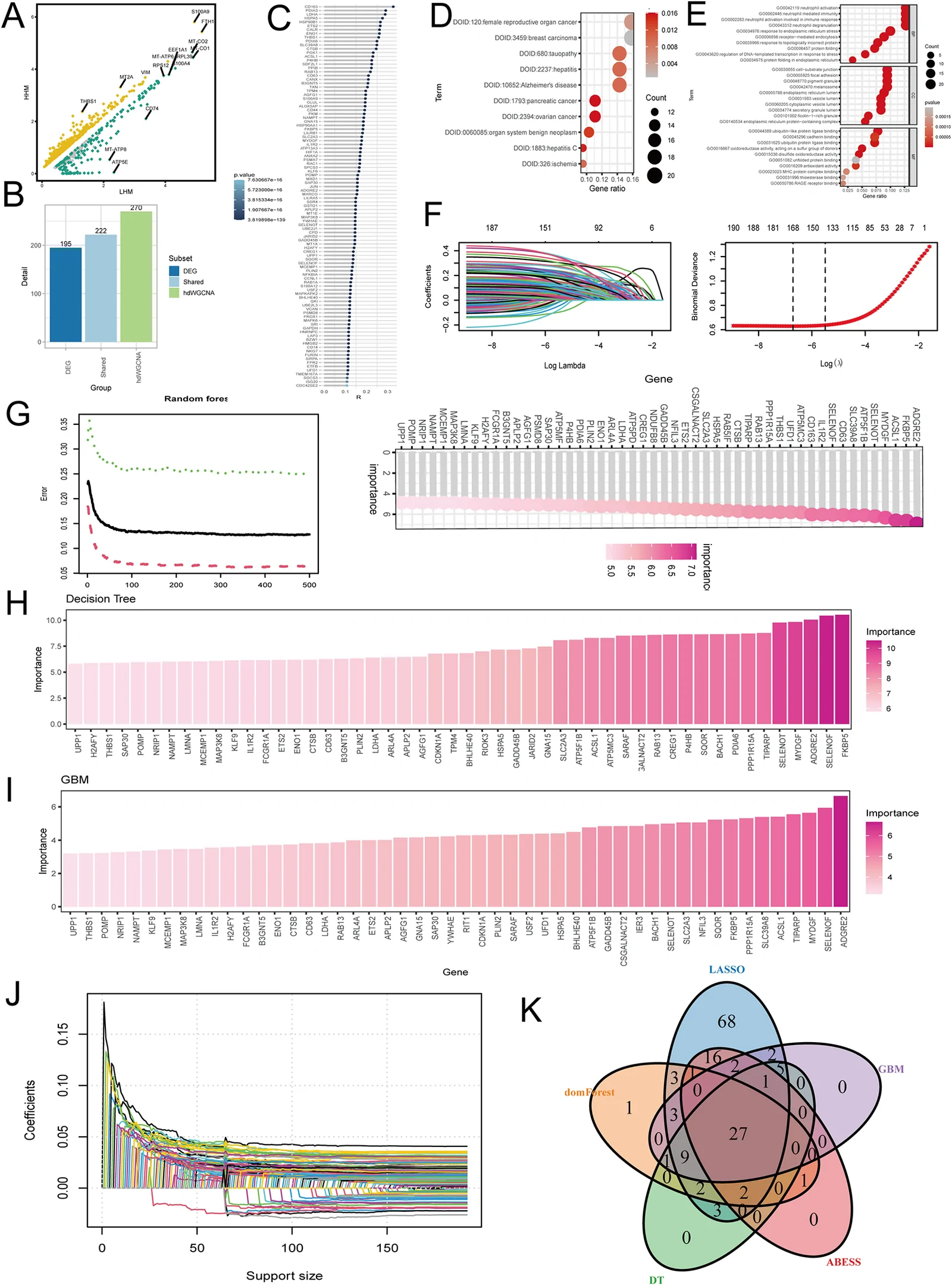
Identification and functional characterization of HRGs in macrophages. **(A)** Volcano plot showing DEGs between HHM and LHM groups, with 417 upregulated genes identified under high hypoxia conditions. **(B)** Venn diagram illustrating the intersection between DEGs and genes from hdWGCNA modules positively correlated with the HHM phenotype, yielding 222 candidate HRGs. **(C)** Pearson correlation analysis identifying 192 genes significantly positively correlated with hypoxia activity in HHM cells. **(D)** Disease Ontology (DO) enrichment analysis highlighting disease associations of HRGs, with a prominent enrichment in hepatitis-related pathways. **(E)** Gene Ontology (GO) enrichment analysis demonstrating significant involvement of HRGs in neutrophil activation and neutrophil-mediated immunity. **(F–J)** Feature selection using multiple machine learning algorithms, including LASSO **(F)** random forest **(G)** decision tree **(H)** gradient boosting machine **(I)** ABESS **(J)**, identifying hub candidate genes with diagnostic potential. **(K)** Venn diagram showing the overlap of selected genes from five machine learning methods, resulting in 27 common hub HRGs.

Subsequently, by utilizing machine learning algorithms, we further filtered out the hub HRGs with significant diagnostic value. Through LASSO analysis, a total of 169 genes were identified (**Figure 6F**; **Supplementary Table 9**). Subsequently, 192 hub genes were identified via RF (**Figure 6G**; **Supplementary Table 10**). Additionally, by using the DT and GBM algorithms, 188 and 146 hub genes were respectively identified (**Figures 6H, I**; **Supplementary Tables 11**, **12**). Moreover, the ABESS algorithm was employed to select another group of 50 hub genes (**Figure 6J**; **Supplementary Table 13**). Finally, through cross-analysis of the feature genes identified by these five machine learning algorithms, 27 common hub genes were determined (**Figure 6K**; **Supplementary Table 14**).

### Evaluation of machine learning benchmark models

The prediction of the AUC diagnostic model indicates that the candidate genes demonstrate relatively consistent and moderately high discriminatory capacity in diagnostic efficacy. Based on the AUC ranking chart in the figure, LDHA, ETS2, FKBP5, ENO1 and CD63 are ranked at the top, with relatively high AUC values (**Figures 7A, B**). The Precision - Recall curve of the training set (N = 11,544) reveals that the model can maintain high precision and recall rates across most threshold ranges, and the model exhibits extremely strong discriminative ability during the training stage. In contrast, the PR curve of the test set (N = 4,947) remains at a relatively high level overall; however, as the recall rate rises, the precision rate gradually declines. The ROC curve also shows good discriminatory efficacy, yet it is slightly lower than that of the development set (**Figure 7C**). Decision curve analysis (DCA) further proves that our model is very useful in clinic, because it gets the highest net income under various probability thresholds (**Figure 7D**). The Recall and ROC curves indicated that the ranger, SVM and LR models exhibited relatively robust performance, characterized by both high sensitivity and excellent accuracy (**Figure 7E**). The box plot revealed that the Mean AUC of each model after repeated validation ranged approximately from 0.78 to 0.92, suggesting that the constructed classifiers generally possess good diagnostic potential. However, the stability and performance levels vary among the models. Among them, the ranger model had the highest median AUC, indicating that it not only demonstrates the best predictive performance but also exhibits good repeatability and robustness (**Figure 7F**). The confusion matrix analysis demonstrated that both the training set and the test cohort displayed good discriminative capabilities (**Figure 7G**). The SHapley Additive exPlanations (SHAP) analysis further showed that TIPARP, ACSL1, PLIN2, FKBP5, ETS2, and B3GNT5 made the most significant contributions to the model output, and their higher overall expression tended to drive the prediction towards the positive category (**Figure 7H**). The correlation scatter plot indicated that the standardized values of most features were positively correlated with the SHAP values, and the slope of the local trend line remained consistent, suggesting that these variables have a stable and consistent promoting effect on the model decision (**Figures 7I, J**). To compare the predictive performance of the different classifiers, we performed repeated five-fold cross-validation with 10 repetitions, yielding 50 matched test folds for each model. The resulting AUC distributions showed distinct performance patterns among the evaluated classifiers, as assessed using the paired Friedman rank test (**Supplementary Figure S2A**). We further quantified the mean AUC differences between the random forest model and each comparator. The corresponding 95% confidence intervals were estimated using the Nadeau–Bengio corrected repeated cross-validation test, with Holm adjustment applied across all 28 pairwise comparisons. Positive AUC differences indicated superior predictive performance of the random forest model relative to the corresponding comparator (**Supplementary Figure S2B**).

**Figure 7:**
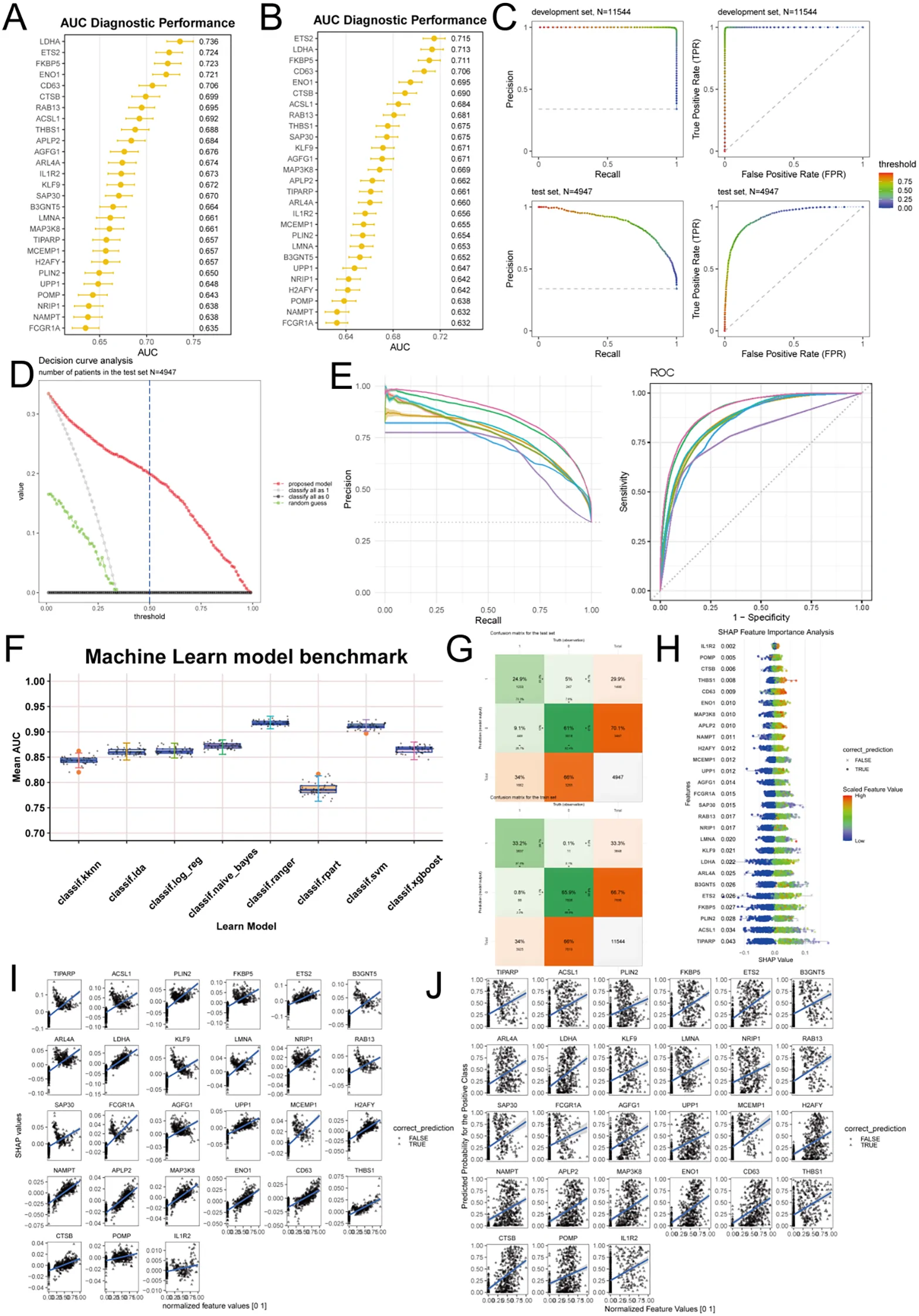
Diagnostic model evaluation and feature contribution analysis demonstrate robust predictive performance. **(A, B)** Ranking of candidate genes based on AUC values. **(C)** PR and ROC curves for the training and test sets. **(D)** DCA demonstrating the clinical utility of the model across a range of probability thresholds. **(E)** Performance comparison of different machine learning models indicating consistently strong sensitivity and accuracy. **(F)** Box plots of AUC values from repeated validation. **(G)** Confusion matrices for the training and test cohorts, confirming good classification performance. **(H)** SHAP analysis identifying hub contributing genes that drive model predictions. **(I, J)** Correlation scatter plots between standardized feature values and SHAP values, indicating consistent positive contributions of most variables to model output.

### Verification of HHM key genes and analysis of drug sensitivity

First, the expression levels of 27 key genes were evaluated in the HC group, Early group, and Late group (**Figure 8A**). Subsequently, the gene TIPARP, which was of interest, was selected for further study. To explore the spatial expression characteristics of TIPARP, its expression density in all single cells was initially visualized (**Figures 8B–D**). When concentrating on macrophages, TIPARP exhibited a more extensive expression (**Figures 8E–G**). Subsequently, we assessed the expression levels of TIPARP in LHM, IHM, and HHM. The expression of TIPARP was notably increased in HHM (**Figure 8H**). Thereafter, we evaluated the expression of TIPARP in all cell types. TIPARP was also significantly elevated in macrophages (**Figure 8I**).

**Figure 8:**
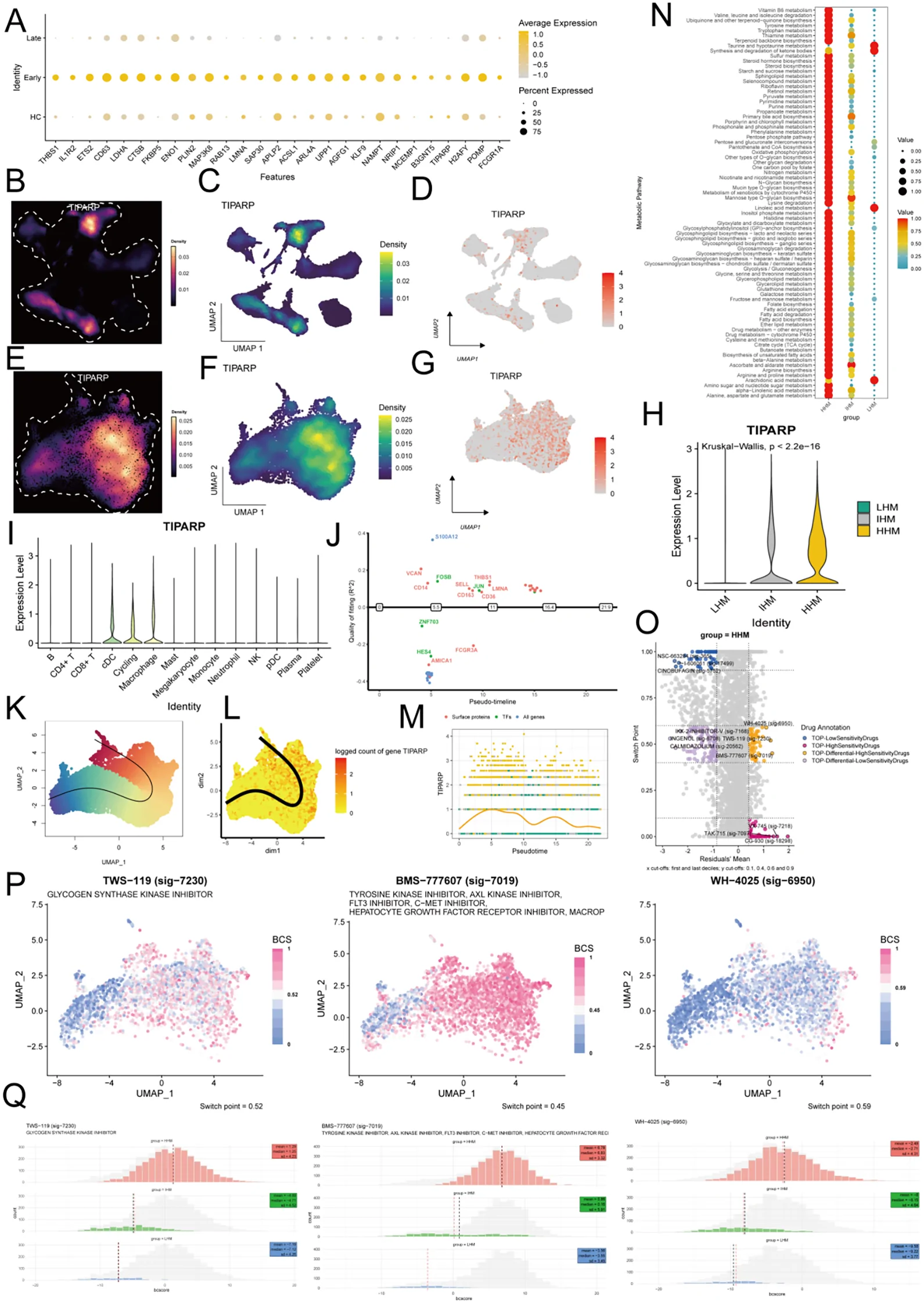
TIPARP expression, trajectory dynamics, metabolic features and drug sensitivity analysis across macrophage subpopulations. **(A)** Expression profiles of 27 key genes across HC, Early and Late groups. **(B–D)** Global visualization of TIPARP expression density across all single cells in the low-dimensional space. **(E–G)** Distribution of TIPARP expression specifically in macrophages. **(H)** Comparative expression of TIPARP among LHM, IHM and HHM. **(I)** TIPARP expression across all cell types, with marked elevation in macrophages. **(J)** Gene expression dynamics along pseudotime. **(K, L)** Trajectory analysis illustrating continuous cellular distribution and branching differentiation paths. **(M)** Feature map showing the spatial distribution of TIPARP expression along the trajectory, with higher expression enriched in intermediate and branching states. **(N)** Bubble plot of metabolic pathway enrichment. **(O)** Drug sensitivity scatter plot based on residual mean and switch point. **(P)** UMAP distribution of drug sensitivity scores. **(Q)** Group-wise comparison of bcscore values.

The results of gene fitting, which vary with the sequence changes, further demonstrate that marker genes such as VCAN, CD14, and S100A12 display significant dynamic fluctuations during the trajectory process. This reflects the transcriptional program alterations of cells during the processes of inflammatory activation, myeloid differentiation, and functional remodeling (**Figure 8J**). The trajectory diagram reveals that cells form a distinct continuous distribution in the two - dimensional embedded space. The black main curve delineates the potential differentiation path from the early state on the left to the branching states on the right and above, indicating clear developmental continuity and branching heterogeneity within this population (**Figures 8K, L**). The feature map in the reduced - dimensional space also indicates that cells with high expression of TIPARP are primarily distributed in the middle of the trajectory and some branching regions, further corroborating its close association with the heterogeneity of macrophage development (**Figure 8M**). The metabolic pathway bubble chart further demonstrates that the HHM group exhibits a higher enrichment level in pathways related to amino acid metabolism, lipid metabolism, sugar metabolism, and exogenous substance metabolism. This finding reveals that the cells in the HHM group display stronger proliferation drive, activation of inflammatory signals, and metabolic adaptability (**Figure 8N**).

Finally, we conducted a further evaluation of the drug sensitivity of the three subgroups. The drug response profile of the HHM group experienced significant remodeling. The overall scatter plot was constructed based on the combined assessment of the Residuals’ Mean and the switch point for drug sensitivity. It indicated that WH - 4025 (sig - 6950), BMS - 777607 (sig - 7019) and TWS - 119 (sig - 7230) were situated in the region of high - differential sensitivity drugs (**Figure 8O**). The UMAP distribution further demonstrated that the high BCS signal coverage of BMS - 777607 was the broadest, nearly encompassing all the main cell populations, with a switch point of 0.45. This suggests that it possesses a strong inhibitory potential for most cell states. TWS - 119 and WH - 4025 also exhibited certain sensitivity, yet their spatial distribution was more heterogeneous (**Figure 8P**). The group histogram revealed that the bcscore of the three candidate drugs all presented an increasing trend of HHM > IHM > LHM (**Figure 8Q**).

### Experimental verification of TIPARP and hypoxia

In order to investigate the relationship between TIPARP and hypoxia-related signaling pathways in sepsis, we first used LPS to stimulate RAW264.7 macrophage cells to establish a cell model, and then detected the expression levels of TIPARP and HIF-1α. Western blot analysis showed that compared with the control group, LPS treatment significantly increased the protein levels of HIF1A and TIPARP (**Figures 9A, B**). Similarly, qRT-PCR analysis indicated that after LPS stimulation, the mRNA expression of HIF1A and TIPARP significantly increased (**Figure 9C**). To determine whether TIPARP is responsive to pathological stress, cells were exposed to hypoxia stimulation. Under hypoxia (2% O_2_), TIPARP protein progressively increased, accompanied by parallel induction of HIF1A and VEGF, with the strongest response observed at later time points (**Figures 9D, E**). Similarly, inflammatory stimulation induced a concentration-dependent increase in TIPARP, HIF1A and VEGF expression (**Figures 9F, G**). Finally, we tested whether TIPARP is necessary for the generation of inflammatory activation under hypoxic conditions. We generated macrophages with sh*TIPARP*, and then we verified the knockdown efficiency of sh*TIPARP*#1 and sh*TIPARP*#2 (**Supplementary Figures S3A, B**). Subsequently, we selected sh*TIPARP*#1 with the highest knockdown efficiency. Under normoxic conditions, the deletion of TIPARP has little effect on the basal HIF1A signaling pathway; however, under hypoxic conditions, the absence of TIPARP significantly increases the accumulation of HIF1A protein and upregulates the expression of the hypoxia response factor BNIP3, compared to the hypoxia control group cells (**Figures 9H, I**). Consistent with the changes at the protein level, qRT-PCR analysis shows that after TIPARP deletion, the expression of HIF1A and VEGF induced by hypoxia is further enhanced (**Figure 9J**). In summary, although TIPARP is activated simultaneously with HIF-1α in response to hypoxia stimulation, its subsequent effect can inhibit the excessive activation of the HIF1A pathway, which supports the view that TIPARP has a negative feedback regulatory function in the cellular hypoxia response.

**Figure 9:**
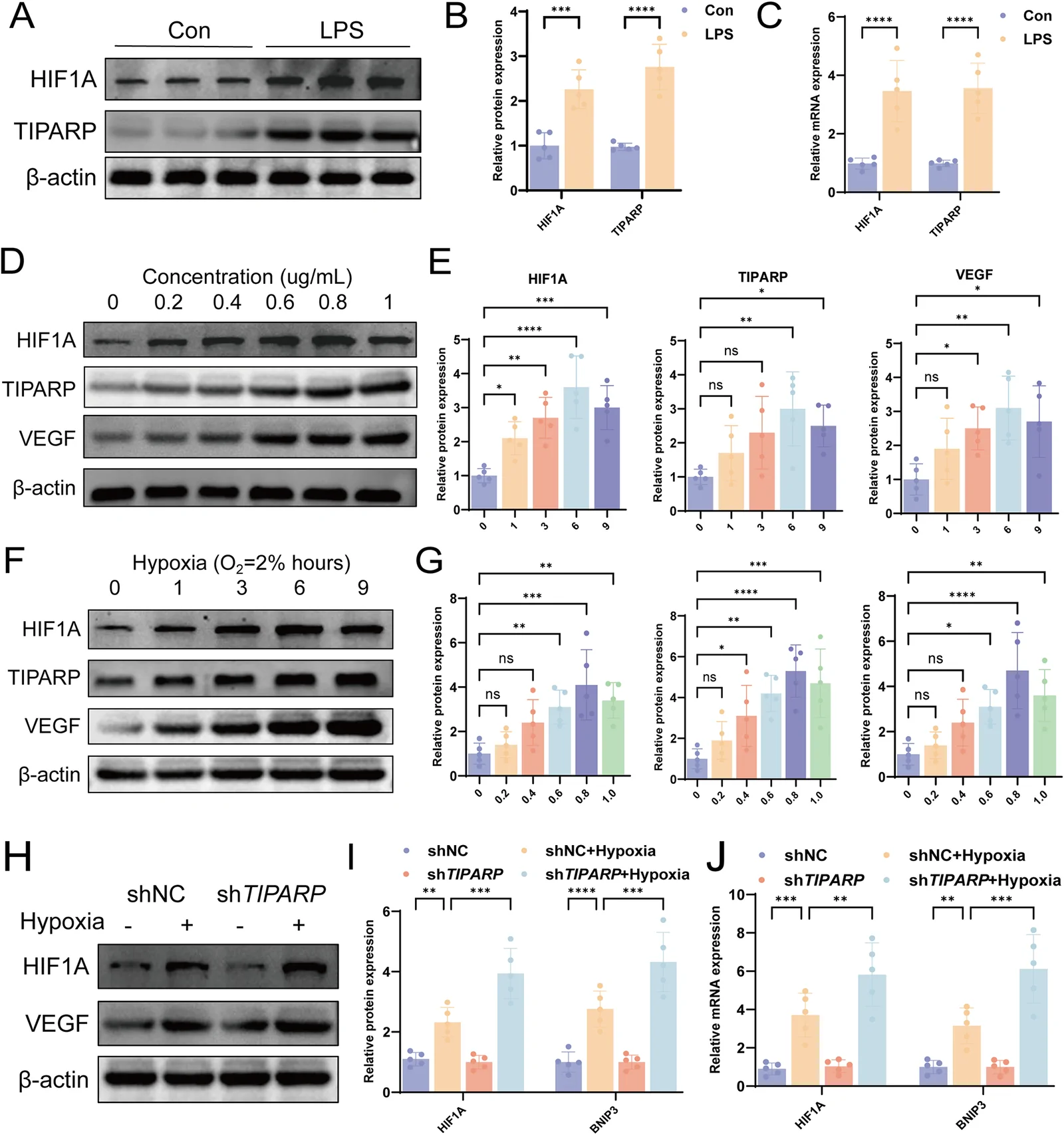
Expression verification of TIPARP and verification of hypoxia. **(A, B)** Western blot analysis and quantification of TIPARP expression in RAW264.7 macrophage cells. **(C)** qRT-PCR analysis of TIPARP mRNA expression in control and patient samples. **(D, E)** Representative western blots and quantification showing time-dependent induction of TIPARP, HIF1A and VEGF under hypoxia (2% O_2_). **(F, G)** Western blot analysis showing dose-dependent upregulation of TIPARP, HIF1A and VEGF following inflammatory stimulation. **(H–J)** Knockdown of TIPARP levels can enhance the expression of HIF1A and VEGF induced by hypoxia. Data are presented as mean ± SEM. *p < 0.05, **p < 0.01, ***p < 0.001, ****p < 0.0001.

## Discussion

Sepsis is increasingly understood as a dynamic immune–metabolic disorder in which inflammation, tissue hypoxia and cellular metabolic rewiring evolve in parallel (11, 29, 30). Rather than representing a passive consequence of impaired perfusion, hypoxia actively reshapes innate immune function through HIF-dependent transcriptional programs (31–33). In sepsis, inflammatory signaling and tissue hypoxia can converge on HIF-1α, which regulates metabolic adaptation and immune responses in innate immune cells, including macrophages and neutrophils. However, the biological consequences of HIF-1α activation may differ according to immune-cell type, tissue context, and disease stage. The enrichment of HIF-1α-related programs observed in high-hypoxia macrophages in the present study is consistent with this sepsis-specific regulatory framework (34–36).

A principal finding of this work is that macrophages exhibit the most pronounced hypoxia-associated transcriptional activity among immune populations in sepsis. This observation is consistent with the recognized role of macrophages as highly plastic sentinels that integrate pathogen-derived signals, oxygen availability and metabolic stress. In sepsis, macrophage polarization is not adequately captured by the conventional M1/M2 framework; instead, macrophages occupy a continuum of activation states shaped by inflammatory ligands, metabolic substrates and tissue microenvironmental cues (37, 38). The stratification of macrophages into low-, intermediate- and high-hypoxic states provides a conceptual advance by positioning hypoxia as an organizing axis of macrophage heterogeneity. Single-cell studies in sepsis have revealed marked immune-cell heterogeneity, temporal remodeling of monocytes/macrophages and disease-stage-specific transcriptional shifts. Our findings extend these reports by showing that hypoxic activity defines functionally distinct macrophage states and may explain part of the transcriptional variability previously observed across septic cohorts (39, 40).

Mechanistically, the high-hypoxic macrophage phenotype appears tightly coupled to inflammatory amplification. HIF1A is essential for myeloid inflammatory responses and supports glycolytic ATP generation under oxygen-limited conditions. Experimental evidence further shows that HIF1α-driven glycolysis promotes macrophage M1-like polarization, whereas macrophage-specific HIF1A activation can enhance NF-κB-associated inflammatory signaling (41, 42). These data support a model in which hypoxia and inflammatory signaling form a feed-forward circuit: inflammatory stimuli stabilize or induce HIF1A, while HIF1A enhances glycolysis and sustains cytokine-producing macrophage states. The enrichment of metabolic pathways in high-hypoxic macrophages is also biologically plausible. Sepsis induces profound immunometabolic remodeling, including altered glycolysis, mitochondrial dysfunction, lactate accumulation, lipid remodeling and amino-acid metabolism. These metabolic changes are not merely adaptive; they can determine whether macrophages promote pathogen clearance, inflammatory injury, immune paralysis or tissue repair. Therefore, the HHM phenotype may represent a metabolically permissive inflammatory state that enables macrophages to persist and signal within oxygen-deprived septic niches.

Another important implication is that high-hypoxic macrophages may act as communication hubs within the septic immune microenvironment. The involvement of ligand–receptor axes such as MIF–CD74/CD44, CCL-family chemokines and IL1-related signaling suggests that these cells may coordinate leukocyte recruitment, antigen-presenting-cell activation and inflammatory propagation (43). MIF–CD74 signaling has been linked to macrophage activation, neutrophil accumulation and inflammatory tissue injury, supporting its relevance to sepsis-associated immune-cell crosstalk (44).

At the gene-regulatory level, the identification of LDHA and ENO1 reinforces the central role of glycolysis in hypoxia-adapted macrophages. LDHA-mediated lactate production and ENO1-associated glycolytic flux are classical downstream features of HIF-driven metabolic conversion (45). FKBP5 and ETS2 may further reflect stress-responsive and transcriptional inflammatory remodeling, suggesting that HHM identity is maintained by both metabolic enzymes and regulatory factors. These findings are consistent with broader evidence that macrophage metabolic reprogramming is a determinant of sepsis severity and organ injury (46).

TIPARP/PARP7 emerges as a particularly compelling candidate regulator. TIPARP is a mono-ADP-ribosyltransferase involved in hypoxia responses, innate immune regulation and transcriptional feedback control (47). Prior work demonstrated that TiPARP can form nuclear condensates and regulate HIF1A stability, while PARP7 has also been implicated in innate immune signaling and mono-ADP-ribosylation-dependent control of interferon pathways (48). These observations provide an external mechanistic rationale for the association between TIPARP, hypoxic macrophage states and HIF1A/VEGF-related signaling.

The predicted drug sensitivity of high-hypoxic macrophages suggests a translational direction. Although computational drug-response inference requires experimental validation, it supports the broader concept that disease-associated macrophage states may harbor targetable vulnerabilities (49). Given that current sepsis management remains largely supportive, interventions aimed at macrophage immunometabolism, HIF signaling, or hypoxia-adaptive inflammatory circuits may offer opportunities for precision immunomodulation.

Several limitations should be acknowledged. First, transcriptional hypoxia scores infer hypoxic activity but do not directly measure oxygen tension. Second, single-cell integration across datasets may introduce batch effects and cohort-dependent bias. Third, the absence of spatial transcriptomics limits the ability to localize HHM within true hypoxic tissue niches. Finally, although TIPARP is strongly implicated, macrophage-specific gain- and loss-of-function models are required to establish causality and define whether TIPARP acts upstream, downstream, or within a feedback loop involving HIF1A.

In summary, this study supports a model in which hypoxia acts as a central determinant of macrophage heterogeneity in sepsis. High-hypoxic macrophages represent a plastic, metabolically rewired and communication-active state that may amplify inflammatory injury while exposing state-specific therapeutic vulnerabilities. By integrating single-cell transcriptomics, trajectory inference, intercellular communication, co-expression network analysis, machine learning and experimental validation, this work provides a framework for understanding hypoxia-driven macrophage reprogramming and highlights TIPARP as a promising candidate regulator for future mechanistic and therapeutic studies.

## Data Availability

The original contributions presented in the study are included in the article/**Supplementary Material**. Further inquiries can be directed to the corresponding author.
